# Neuroestradiol regulation of ventromedial hypothalamic nucleus 5′-AMP-activated protein kinase activity and counterregulatory hormone secretion in hypoglycemic male versus female rats

**DOI:** 10.3934/Neuroscience.2021006

**Published:** 2020-12-16

**Authors:** MD Main Uddin, Karen P Briski

**Affiliations:** School of Basic Pharmaceutical and Toxicological Sciences, College of Pharmacy, University of Louisiana Monroe, Monroe, LA 71201, USA

**Keywords:** letrozole, aromatase, insulin-induced hypoglycemia, ventromedial hypothalamic nucleus, AMPK, sex differences

## Abstract

Hypoglycemia activates the ultra-sensitive energy gauge 5′-AMP-activated protein kinase (AMPK) in ventromedial hypothalamic nucleus (VMN) gluco-regulatory neurons. The VMN is exemplified by high levels of expression of the enzyme aromatase, which converts testosterone to estradiol. This study examined the hypothesis that neuroestradiol imposes sex-dimorphic control of VMN AMPK activity during eu- and/or hypoglycemia. VMN tissue corresponding to distinct rostro-caudal segments was obtained by micropunch dissection from testes-intact male and estradiol-replaced ovariectomized female rats that were infused intracerebroventricularly with the aromatase inhibitor letrozole (Lz) before subcutaneous insulin (INS) injection. In euglycemic rats, Lz treatment elevated (male) or decreased (female) middle VMN phosphoAMPK content, with concurrent effects on total AMPK expression. Lz prevented hypoglycemic up-regulation of the mean pAMPK/AMPK ratio in rostral and middle segments of the male VMN, and significantly inhibited this proportion throughout the VMN of hypoglycemic female rats. Lz prevented glucagon secretion in hypoglycemic rats of each sex, and abolished hypoglycemic hypercorticosteronemia in males. Results show that neuroestradiol regulation of VMN AMPK activity during euglycemia is region-specific and gender-divergent, e.g. inhibitory in males versus stimulatory in females. Intra-VMN distribution of hypoglycemia-activated AMPK varies between sexes, but in each sex, locally-generated estradiol is critical for sensor reactivity to this stimulus. Coincident Lz attenuation of VMN AMPK and counter-regulatory hormone responses to hypoglycemia infers a possible cause-and-effect association. Further effort is needed to elucidate the cellular and molecular mechanisms that underlie sex-dimorphic neuroestradiol control of VMN total AMPK and phosphoAMPK expression during distinct metabolic states.

## 1. Introduction

The ultrasensitive, evolutionarily-conserved energy gauge 5′-AMP-activated protein kinase (AMPK) is activated via phosphorylation when cellular AMP levels increase relative to ATP. Hypothalamic AMPK provides crucial input on brain cell ATP availability to neural pathways that govern whole-body energy stability [1]–[3]. There, AMPK integrates diverse metabolic and endocrine indicators of energy paucity (ghrelin, corticosterone, thyroxine, adiponectin) or excess (glucose, leptin, insulin) [4] to regulate food intake, glucose homeostasis, energy expenditure, and body weight [5]. Activation of mediobasal hypothalamic (MBH) AMPK is obligatory for optimum glucose counter-regulatory responses to insulin-induced hypoglycemia [6],[7]. The ventromedial hypothalamic nucleus (VMN), a prominent neuroanatomical component of the MBH, is a likely source of AMPK gluco-regulatory signaling as hypoglycemia increases AMPK phosphorylation in VMN neurons that express characterized gluco-inhibitory (γ-aminobutyric acid; GABA) or stimulatory (nitric oxide; NO) neurotransmitters [8],[9].

The VMN is a substrate for estradiol regulation of circulating glucose levels [10],[11]. In the VMN and elsewhere in the brain, estrogen receptors (ERs) are stimulated by estradiol of dual origin, as this hormone is both acquired from the circulation and produced locally by aromatase conversion of testosterone to estradiol. Brain tissue aromatase protein levels vary according to structure, with highest expression levels observed in the VMN and a limited number of other sites [12]–[14]. The impact of neuroestradiol on VMN AMPK activity has not been established for either sex. In the current study, methodology for intracerebroventricular (*icv*) infusion of the aromatase inhibitor letrozole (LZ) was used in conjunction with high-resolution microdissection/Western blot techniques in a rat whole-animal model to investigate the premise that neuroestradiol exerts sex-dimorphic control of VMN AMPK protein expression and/or activation and counter-regulatory hormone secretion during systemic glucose availability or scarcity. Our studies show that in each sex, neuroestradiol regulates VMN gluco-regulatory transmission in a region-specific manner during hypoglycemia [15]. Current work analyzed total AMPK as well as activated, e.g. phosphoAMPK (pAMPK) expression in micropunch-dissected tissue samples obtained from rostral, middle, and caudal levels of the VMN to determine if aromatase-dependent hypoglycemic activation of AMPK is co-localized with neuroestradiol-sensitive metabolic transmitters in this structure.

## 2. Materials and methods

### 2.1. Animals

Adult male and female Sprague Dawley rats (3–4 months of age) were housed in groups of 2–3 according to sex, under a 14 h light/10 h dark light cycle (lights on at 05.00 h). Animals had unrestricted access to standard laboratory chow and water, and were acclimated to daily handling. All surgical and experimental protocols were performed in compliance with NIH Guidelines for the Care and Use of Laboratory Animals, 8^th^ Edition, with approval by the ULM Institutional Animal Care and Use Committee.

### 2.2. Experimental design

**Figure 1.**
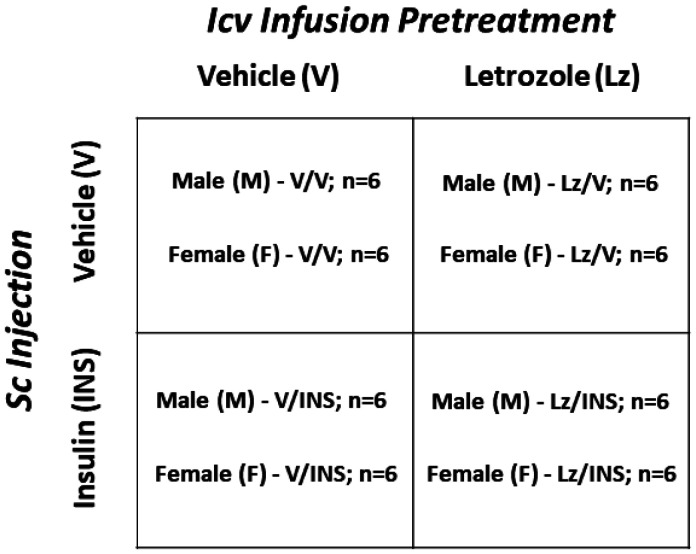
Experimental Design. Using a 2 × 2 × 2 block design, groups of adult testes-intact male (M) and ovariectomized, estradiol-implanted female (F) rats were infused into the left lateral cerebral ventricle, via Alzet pump (Model 1007 D; 0.5 uL/hour), with the aromatase enzyme inhibitor letrozole (Lz; 1.67 µg/µL) or vehicle [V; 30% artificial cerebrospinal fluid (aCSF)/70% dimethyl sulfoxide (DMSO)] from study day 1 through 6. On study day six, groups of Lz- or V-infused animals of each sex were injected subcutaneously (*sc*) at 9.00 hr with neutral protamine Hagedorn insulin (INS; 10.0 U/kg bw) or vehicle; at 10.00 hr on the same day, animals were sacrificed for brain and trunk blood collection.

Animals of each sex were divided into four treatment groups (Figure 1). Five days before experimentation, female rats were bilaterally ovariectomized (OVX) and implanted with a *sc* silastic capsule (*i.d*. 0.062 inch, *o.d*. 0.125 inch; 10 mm/100 g *bw*) filled with 30 µg 17β-estradiol-3-benzoate/mL safflower oil to reproduce circulating steroid levels measured in ovary-intact metestrus female rats [16]. On study day 1, male and female animals were implanted with a 28-gauge stainless-steel cannula (Alzet Brain Infusion Kit 1, DURECT™ Corporation, Cupertino, CA) into the left lateral ventricle (LV) [coordinates: 0.0 mm posterior to *bregma*; 1.5 mm lateral to *bregma*; 3.5 mm ventral to brain surface] [8], connected to a primed Model 1007D Alzet osmotic minipump (0.5 uL/hour) containing vehicle (V; 30% artificial cerebrospinal fluid (aCSF)/70% dimethyl sulfoxide (DMSO) or Lz (1.67 µg/µL [17]; prod. no. L0248; Tokyo Chemical Industries, Tokyo, Japan). After surgery, animals were injected with ketoprofen (1 mg/kg bw *sc*) and enrofloxacin (10 mg/0.1 mL IM), and transferred to individual cages. At 9.00 hr on study day 6, animals were injected *sc* with sterile diluent [V; Eli Lilly & Co., Indianapolis, IN; male groups 1 (M-V/V; n = 6) and 3 (M-Lz/V; n = 6); female groups 1 (F-V/V; n = 6) and 3 (F-Lz/V; n = 6)] or neutral protamine Hagedorn insulin (INS; 10.0 U/kg bw; Henry Schein [18]; male groups 2 (M-V/INS; n = 6) and 4 (M-Lz/INS; n = 6); female groups 2 (F-V/INS; n = 6) and 4 (F-Lz/INS; n = 6). At 10.00 hr on day 6, animals were anesthetized with isoflurane for blood collection by cardiac puncture, then sacrificed by microwave fixation (1.45 sec; In Vivo Microwave Fixation System, 4.5kW; Stoelting Co., Wood Dale, IL). Brains were snap-frozen in a liquid nitrogen-cooled isopentane and stored at −80 °C. Plasma was stored at −20 °C.

### 2.3. VMN tissue micropunch dissection and Western Blot analysis

**Figure 2.**
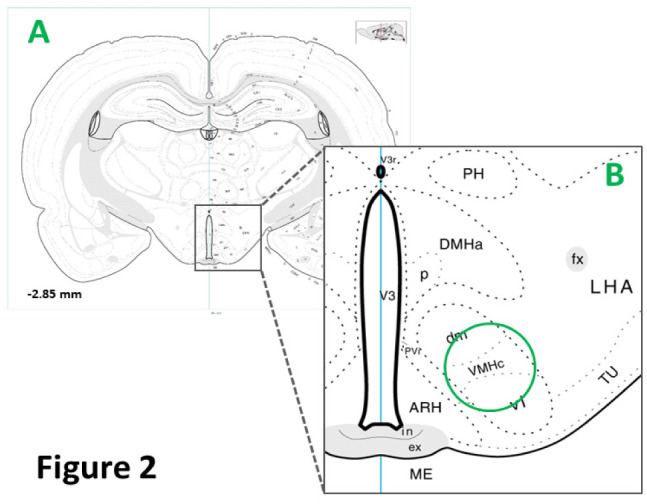
Ventromedial Hypothalamic Nucleus (VMN) Micropunch Dissection. The rectangle in the Panel A brain map (−2.85 mm posterior to *bregma*) depicts the VMN within the mediobasal hypothalamus, and is enlarged (Panel B) to illustrate the location of VMN in that region. The blue circle denotes positioning of a 0.50 mm diameter circular micropunch tool over the center of the elliptical-shaped VMN, enabling sampling of tissue from dorsomedial, central, and ventrolateral divisions of the VMN. Abbreviations in Panel B: ARH: arcuate hypothalamic n.; DMHa p: anterior, posterior dorsomedial hypothalamic n.; fx: fornix; LHA: lateral hypothalamic area; ME: median eminence; PVi: intermediate periventricular hypothalamic n.; VMHc, dm, vl: central, dorsomedial, ventormlateral ventromedial hypothalamic n.; TU: tuberal n.; V3: third ventricle.

Each brain was cut into consecutive 100 µm-thick frozen sections through the VMN between −2.00 and −3.3 mm posterior to *bregma*. For each animal, bilateral micropunches of VMN tissue were taken using a calibrated 0.5 mm hollow punch tool (prod. no. 57401; Stoelting Co., Kiel, WI), as shown in Figure 2, from sections cut at rostral (−2.0 to −2.3 mm), middle (−2.5 to −2.8), and caudal (−3.0 to −3.3 mm) levels of the VMN, and pooled according to region in lysis buffer [18] for Western blot analysis. Accuracy of use of micropunch methodology for collection of distinctive hypothalamic loci of interest, including the VMN, as indicated by marker protein expression, has been verified [19]. In each treatment group, tissue lysate aliquots from individual subjects were combined for rostral, middle, and caudal VMN to create six separate sample pools for each protein of interest for each VMN segment. Tissue sample pools were separated in Bio-Rad TGX 10% stain-free gels (Bio-Rad, Hercules, CA); after electrophoresis, gels were UV light-activated (1 min) in a Bio-Rad ChemiDoc TM Touch Imaging System [20] and proteins were transblotted to 0.45 µm PVDF-Plus membranes (prod. no. 121639; Data Support Co., Panorama City, CA). Membranes were blocked with Tris-buffer saline containing 0.1% Tween-20 and 2.0% bovine serum albumin prior to incubation between 36–42 h (4 °C) with primary polyclonal antisera raised in rabbit against total (activated and inactivated) AMPK_α1/2_ enzyme protein (prod. no. 2532; 1:2,000; Cell Signaling Technology, Danvers, MA) or the active enzyme form, e.g. phosphorylated AMPK_α1/2_-Thr 172 (pAMPK; prod. no. 2535; 1:2,000; Cell Signaling Technol). Membranes were then incubated with a goat anti-rabbit horseradish- peroxidase-labeled secondary antiserum (1:5,000; prod. no. NEF812001EA; PerkinElmer, Waltham, MA), followed by SuperSignal West Femto maximum sensitivity chemiluminescence substrate (prod. no. 34096; ThermoFisherScientific, Waltham, MA). Automated membrane buffer washes and blocking and antibody incubations were performed in a Freedom Rocker™ Blotbot. Protein band optical density (O.D.) measures were normalized to total in-lane protein using Image Lab™ 6.0.0 software (Bio-Rad). Precision plus protein molecular weight dual color standards (prod. no. 161–0374, Bio-Rad) were included in each Western blot analysis.

### 2.4. Plasma glucose and hormone analyses

Circulating glucose levels was measured with an ACCU-CHECK Aviva Plus glucometer (Roche Diagnostics USA, Indianapolis, IN) [21]. Circulating corticosterone (ADI-900-097; Enzo Life Sciences, Inc., Farmingdale, NY) and glucagon (EZGLU-30K, EMD Millipore, Billerica, MA) concentrations were determined using commercial ELISA kit reagents, as described [22].

### 2.5. Statistical analyses

Mean normalized VMN protein O.D. and plasma glucose and hormone data were evaluated by three-way analysis of variance and Student-Newman-Keuls *post-hoc* test. Differences of p < 0.05 were considered significant. In each figure, statistical differences between specific pairs of treatment groups are denoted as follows: * p < 0.05; ** p < 0.01; *** p < 0.001; **** p < 0.0001.

## 3. Results

**Figure 3.**
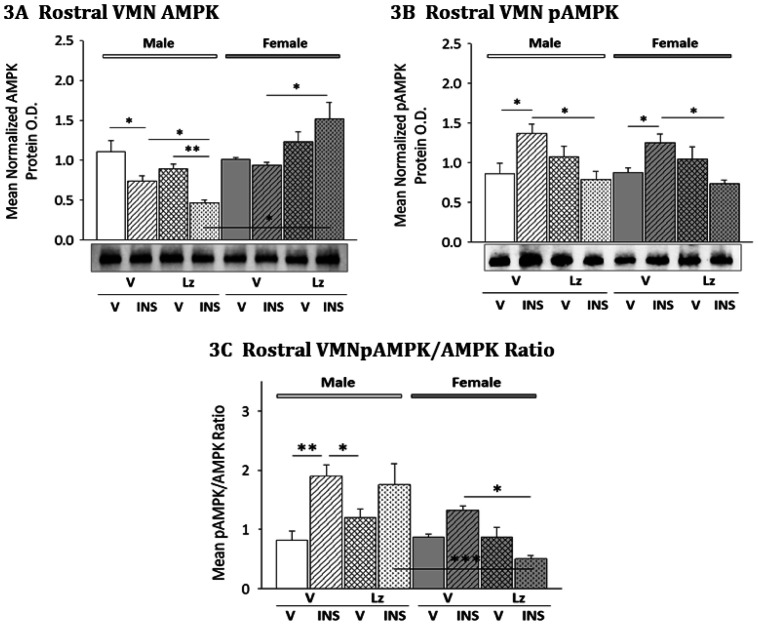
Effects of Intracerebroventricular (*icv*) Lz Administration on Rostral Ventromedial Hypothalamic Nucleus (VMN) 5′-AMP-Activated Protein Kinase (AMPK) and PhosphoAMPK (pAMPK) Protein Expression in Eu- versus Hypoglycemic Male and Female Rats. VMN tissue from groups of Lz- or V-pretreated, INS- or V-injected male and female rats was collected by bilateral micropunch dissection from frozen tissue section cut between −2.00 and −2.30 mm posterior to bregma. Data show mean normalized rostral VMN AMPK (Figure 3A; F_(7,40)_ = 9.07; p < 0.0001; sex main effect: F_(1,40)_ = 25.26; p < 0.0001; sex/pretreatment interaction: F_(1,40)_ = 18.66; p < 0.0001; sex/injection interaction: F_(1,40)_ = 11.60; p = 0.002) and pAMPK (Figure 3B; F_(7,40)_ = 4.02; p = 0.005; pretreatment main effect: F_(1,40)_ = 5.01; p = 0.035; pretreatment/injection interaction: F_(1,40)_ = 21.61; p < 0.0001) optical density (O.D.) measures + S.E.M. for groups of male (M; *left-hand* side; solid or patterned white bars) and female (F; *right-hand* side; solid or patterned gray bars) rats treated as follows: V infusion/V injection (M-V/V, solid white bars, n = 6; F-V/V, solid gray bars; n = 6), V infusion/INS injection (M-V/INS; diagonal-striped white bars; n = 6; F-V/INS; diagonal-striped gray bars; n = 6), Lz infusion/V injection (M-Lz/V; cross-hatched white bars; n = 6; F-Lz/V); cross-hatched gray bars, n = 6), Lz infusion//INS injection (M-Lz/INS; stippled white bars; n = 6; F-Lz/INS; stippled gray bars; n = 6). Figure 3C illustrates mean rostral VMN pAMPK/AMPK ratio (F_(7,40)_ = 7.69; p < 0.0001; sex main effect: F_(1,40)_ = 18.17; p < 0.0001; injection main effect: F_(1,40)_ = 12.01; p = 0.002; sex/injection: F_(1,40)_ = 9.82; p = 0.005; pretreatment/injection interaction: F_(1,40)_ = 7.50; p = 0.011). * p < 0.05; ** p < 0.01; *** p < 0.001; **** p < 0.0001.

Male and female rats were infused *icv* with the aromatase enzyme inhibitor letrozole (Lz) prior to *sc* vehicle (V) or insulin (INS) injection to investigate the impact of neuroestradiol on rostral VMN AMPK and pAMPK protein expression during eu- or hypoglycemia (Figure 3). Data presented in Figure 3A show that INS injection significantly decreased AMPK expression in males [M-V/INS (diagonal-striped white bar) versus M-V/V (solid white bar)], but not females. Lz infusion did not alter AMPK profiles in V-injected animals of either sex. In males, Lz pretreatment did not prevent suppression of AMPK levels by INS [M-Lz/INS (stippled white bar) versus M-Lz/V (cross-hatched white bar)]. In females, Lz pretreatment elevated AMPK expression during hypoglycemia [F-Lz/INS (stippled gray bar) versus F-V/INS (diagonal-striped gray bar)].

Effects of *icv* Lz administration on rostral VMN pAMPK expression in V- or INS-injected male and female rats are illustrated in Figure 3B. INS injection significantly increased pAMPK expression in male [M-V/INS versus M-V/V] and female [F-V/INS versus F-V/V] rats. Lz infusion did not modify rostral VMN pAMPK content in euglycemic animals of either sex, but prevented hypoglycemic stimulation of this protein profile in both males [M-Lz/INS versus M-Lz/V] and females [F-Lz/INS versus F-Lz/V]. As shown in Figure 3C, Lz prevented augmentation of the mean ratio of pAMPK/AMPK expression in the rostral VMN of male rats, and caused significant diminution of this ratio in the same segment in the female.

**Figure 4.**
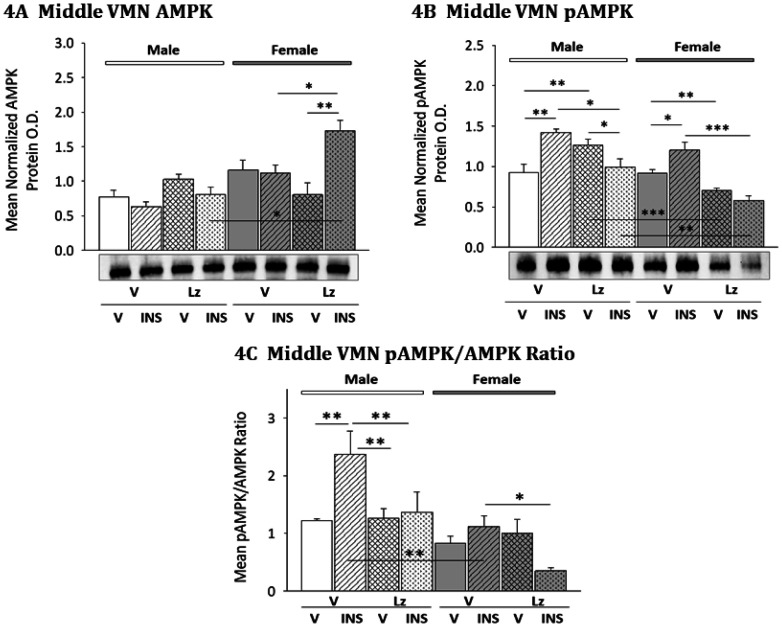
Aromatase Regulation of Middle VMN AMPK and pAMPK Protein Expression in Vehicle- and Insulin-Injected Male and Female Rats. Data illustrate mean normalized middle VMN segment (−2.5 to −2.8 posterior to bregma) AMPK (Figure 4A; F_(7,40)_ = 8.05; p < 0.0001; sex main effect: F_(1,40)_ = 21.07; p < 0.0001; sex/injection interaction: F_(1,40)_ = 13.01; p = 0.001; pretreatment/injection interaction: F_(1,40)_ = 6.58; p = 0.017; sex/pretreatment/injection interaction: F_(1,40)_ = 9.18; p = 0.006) and pAMPK (Figure 4B; F_(7,40)_ = 14.75; p < 0.0001; sex main effect: F_(1,40)_ = 32.93; p < 0.0001; pretreatment main effect: F_(1,40)_ = 19.94; p < 0.0001; sex/pretreatment interaction: F_(1,40)_ = 12.68; p = 0.002; pretreatment/injection interaction: F_(1,40)_ = 31.43; p < 0.0001) O.D. values + S.E.M. are presented for V/V, V/INS, Lz/V, and Lz/INS groups of male (M; *left-hand* side) and female (F; *right-hand* side) rats; n = 6 animals per group. Figure 4C illustrates mean middle VMN pAMPK/AMPK ratio (F_(7,40)_ = 6.38; p < 0.0001; sex main effect: F_(1,40)_ = 20.48; p < 0.0001; pretreatment main effect: F_(1,40)_ = 5.86; p = 0.024; sex/injection interaction: F_(1,40)_ = 6.36; p = 0.019; pretreatment/injection interaction: F_(1,40)_ = 9.69; p = 0.005). * p < 0.05; ** p < 0.01; *** p < 0.001; **** p < 0.0001.

Data presented in Figure 4A show that in both male and female rats, middle VMN AMPK content was unaffected by either INS or Lz treatment alone. In the female, Lz infusion prior to INS injection resulted in significant enhancement of middle VMN AMPK expression [F-Lz/INS versus F-V/INS]. Patterns of middle VMN pAMPK protein expression in male versus female rats injected with INS after *icv* Lz or V infusion are depicted in Figure 4B. Data reveal that INS injection stimulated pAMPK levels in both males and females. Lz infusion significantly elevated or decreased pAMPK expression in euglycemic male [M-Lz/V versus M-V/V] and female [F-Lz/V versus F-V/V] rats, respectively. In each sex, animals pretreated with Lz to hypoglycemia did not exhibit augmentation of middle VMN pAMPK content as observed in V-infused INS-injected animals. Data presented in 4C show that Lz abolished hypoglycemia up-regulation of the mean middle VMN pAMPK/AMPK ratio in male rats, and reduced this ratio in females.

**Figure 5.**
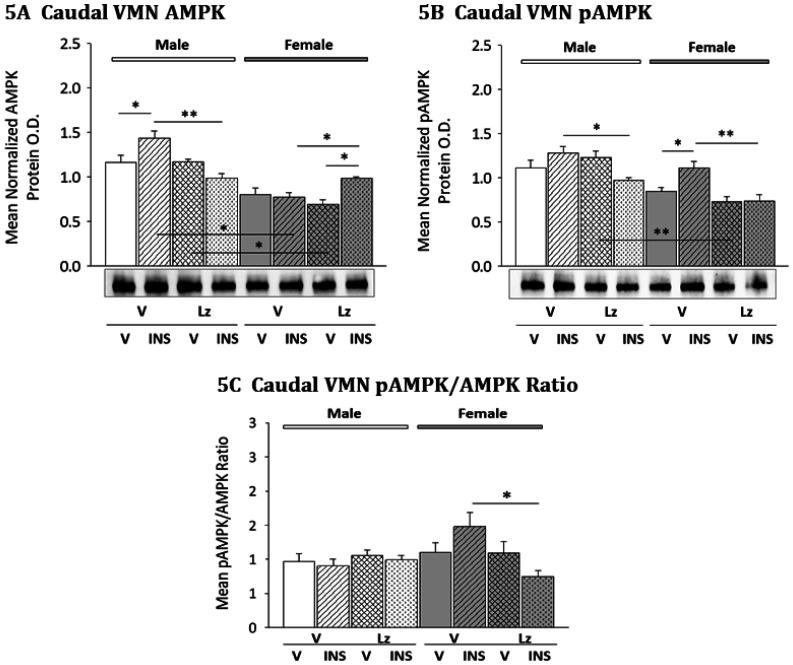
Patterns of Caudal VMN AMPK and pAMPK Protein Expression in Eu- versus Hypoglycemia Male and Female Rats; Effects of *Icv* Lz Infusion. Data illustrate mean normalized caudal VMN segment (−3.0 to −3.3 mm posterior to *bregma*) AMPK (Figure 5A; F_(7,40)_ = 18.44; p < 0.0001; sex main effect: F_(1,40)_ = 84.94; p < 0.0001; sex/pretreatment interaction: F_(1,40)_ = 11.10; p = 0.003; pretreatment/injection interaction: F_(1,40)_ = 31.43; *p* < 0.0001; sex/pretreatment/injection interaction: F_(1,40)_ = 22.35; p < 0.0001) and pAMPK (Figure 5B; F_(7,40)_ = 9.90; p < 0.0001; sex main effect: F_(1,40)_ = 36.60; p < 0.0001; pretreatment main effect: F_(1,40)_ = 12.65; p = 0.002; pretreatment/injection interaction: F_(1,40)_ = 12.38; p = 0.002) O.D. values + S.E.M. are presented for V/V, V/INS, Lz/V, and Lz/INS groups of male (M; *left-hand* side) and female (F; *right-hand* side) rats; n = 6 animals per group. Figure 5C illustrates mean caudal VMN pAMPK/AMPK ratio (F_(7,40)_ = 6.38; p < 0.0001; sex/pretreatment interaction: F_(1,40)_ = 6.27; *p* = 0.019. * p < 0.05; ** p < 0.01; *** p < 0.001; **** p < 0.0001.

Effects of *icv* Lz on caudal VMN AMPK and pAMPK expression in eu- or hypoglycemic male and female rats are presented in Figure 5. As shown in Figure 5A, total AMPK protein was increased by INS in male [M-V/INS versus M-V/V], but not female rats. In males, hypoglycemic stimulation of AMPK profiles was prevented by Lz [M-Lz/INS versus M-Lz/V]. In the female, Lz infusion resulted in a significant increase in caudal VMN AMPK levels in hypoglycemic female rats [F-Lz/INS versus F-V/INS]. Data in Figure 5B indicate that caudal VMN pAMPK levels were elevated by INS injection in female [F-V/INS versus F-V/V], but not male rats. In the female, hypoglycemic stimulation of pAMPK expression was averted by Lz pretreatment [F-Lz/INS versus F-Lz/V]. As illustrated in Figure 5C, Lz had no significant effect on the caudal VMN pAMPK/AMPK protein ratio in the male, but suppressed this ratio in female rats.

**Figure 6.**
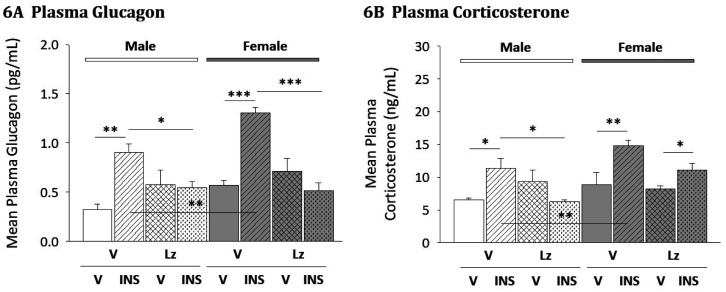
Aromatase Regulation of Counterregulatory Hormone Secretion in Male and Female Rats. Data depict effects of Lz infusion prior to V- or INS-injection of male and female rats on plasma glucagon (Figure 6A; F_(7,40)_ = 11.11; p < 0.0001; sex main effect: F_(1,40)_ = 8.34; p = 0.006; pretreatment main effect: F_(1,40)_ = 8.75; p = 0.005; injection main effect: F_(1,40)_ = 18.05; p < 0.0001; sex/pretreatment interaction: F_(1,40)_ = 4.44; p = 0.04; pretreatment/injection interaction: F_(1,40)_ = 36.64; p < 0.0001) and corticosterone (Figure 6B; F_(7,40)_ = 5.81; p = 0.001; sex main effect: F_(1,40)_ = 8.32; p = 0.008; injection main effect: F_(1,40)_ = 10.32; p = 0.004; pretreatment/injection interaction: F_(1,40)_ = 10.77; p = 0.003) profiles. Bars indicate mean plasma hormone values + S.E.M. for V/V, V/INS, Lz/V, and Lz/INS groups of male (M; *left-hand* side) and female (F; *right-hand* side) rats; n = 6 animals per group. * p < 0.05; ** p < 0.01; *** p < 0.001; **** p < 0.0001.

Lz was administered by *icv* infusion before *sc* V or INS injection to evaluate neuroestradiol regulation of plasma glucose (Table 1) and counter-regulatory hormone (Figure 6) profiles in each sex. Data in Table 1 show that in male and female, INS administration resulted in equivalent reductions in circulating glucose levels [F_(7/40)_ = 68.61; p < 0.0001; injection main effect: F_(1,40)_ = 460.09; p < 0.0001] in V- versus Lz-infused animals. As shown in Figure 6A, plasma glucagon levels were significantly elevated in hypoglycemia male [M-V/INS versus M-V/V] and female [F-V/INS versus F-V/V] rats. Lz infusion did not alter mean glucagon levels in euglycemic animals, but prevented INS-induced increases in hormone secretion [M-Lz/INS versus M-Lz/V; F-Lz/INS versus F-Lz/V]. Data in Figure 6B illustrate hypoglycemic stimulation of corticosterone release in male and female rats. In each sex, Lz had no effect on this hormone profile in V-injected animals, but abolished INS-induced augmentation of corticosterone secretion in males, but not females.

**Table 1.** Effects of letrozole (Lz) pretreatment on plasma glucose levels in vehicle (V)- or insulin (INS)-injected male and female rats.

Sex	Treatment Groups^e^			
	V/V^a^	V/INS^b^	Lz/V^c^	Lz/INS^d^
Male	174.5 ± 11.1	75.3 ± 4.1*	182.3 ± 8.9	72.8 ± 2.3*
Female	169.7 ± 5.9	60.3 ± 3.8*	148.2 ± 7.1***	79.5 ± 4.5**

Note: ^**a**^ intracerebroventricular (*icv*) V infusion days 1–6; subcutaneous (*sc*) V injection day 6; **^b^** *icv* V infusion days 1–6; *sc* neutral protamine Hagedorn insulin (INS; 10 U/kg bw) day 6; **^c^** *icv* letrozole (Lz; 1.67 µg/µL dosage, 0.5 µL/hr infusion rate) infusion days 1–6; *sc* V injection day 6; **^d^** *icv* Lz infusion days 1–6; *sc* INS injection day 6; **^e^** F_(7/40)_ = 68.61; p < 0.0001; * p < 0.0001 versus V-injected controls; ** p < 0.005 versus V-injected controls; *** p < 0.001 M-Lz/V-M versus F-Lz/V.

## 4. Discussion and conclusion

VMN AMPK is an evident source of metabolic-sensory input to the neural glucostatic network, as hypoglycemia activates this sensor in resident neurons that express neurotransmitters involved in glucose counter-regulation. The VMN is a substrate for estradiol input to that neural circuitry [10]. Evidence for high VMN aromatase expression levels raises the prospect that neuroestradiol may govern local AMPK function. Current studies investigated the hypothesis that aromatase may control VMN AMPK activity during glucose homeostasis and/or hypoglycemia in one or both sexes. Data show that in euglycemic rats, neuroestradiol imposes inhibitory or stimulatory control of mid-level VMN pAMPK expression, according to sex. During hypoglycemia, aromatase activity is a negative stimulus for total AMPK protein content over the length of the female VMN, reflected by diminution of the mean pAMPK/AMPK ratio in each segment. In hypoglycemic males, on the other hand, aromatase increases this ratio through augmentation of pAMPK protein expression. Results show that neuroestradiol signaling is requisite for hypoglycemic hyperglucagonemia in each sex and for elevated corticosterone secretion in hypoglycemic males. Ongoing research seeks to establish whether observed diminution of counter-regulatory outflow in Lz-treated animals is a consequence of drug suppression of hypoglycemic activation of VMN AMPK. Present outcomes emphasize the need for clarification of mechanisms that cause sex-dimorphic aromatase regulation of VMN AMPK activity during euglycemia, and those that underlie hypoglycemia-associated adjustments in neuroanatomical extent (both sexes) and direction (males) of neuroestradiol control of VMN sensor activation.

Data here provide novel evidence that aromatase regulates baseline AMPK activity within a distinct rostro-caudal segment of the male and female VMN, where direction of control is sex-specific. As ERs occur over the length of the VMN in each sex, it is unclear how this regulatory action is confined to the mid-VMN. A conceivable explanation is that aromatase production and/or activity is elevated in that segment compared to others, resulting in relatively higher neuroestradiol tissue levels. Conversely, neuroestradiol yield may be equivalent throughout the VMN, but input via ERs may vary by region due to dissimilar magnitude of ER expression or post-receptor signaling in AMPK-expressing neurons. Since basal middle VMN aromatase protein profiles are similar in the two sexes [15], bi-directional effects of neuroestradiol on pAMPK expression in that location, e.g. inhibition in males versus augmentation in females could reflect, in part, differences in absolute and proportional expression of ER variants, including classical (ER-alpha and ER-beta) and membrane (G protein-coupled ER-1; GPER/GPR30) ERs, that may regulate AMPK activity in that location. This notion remains speculative until verified by additional research. Lz administration did not affect baseline plasma glucose or counter-regulatory hormone profiles despite adjusted VMN pAMPK expression, which infers that sensor activity in the mid-VMN does not regulate glucostasis, or alternatively, that altered metabolic sensory signaling in that site does not override metabolic cues that are unaffected by this *icv* drug treatment.

Implementation here of an *icv* route of Lz administration raises the possibility that demonstrable drug effects on VMN AMPK activity may result from, to some extent, diminished aromatase activity within and outside the VMN. Indeed, the VMN is extensively interconnected with other forebrain structures that participate in gluco-regulation, including the lateral hypothalamic area and arcuate, dorsomedial, and paraventricular hypothalamic nuclei. However, evidence for high VMN aromatase expression profiles supports the view that treatment outcomes are largely reflective of Lz inhibition of local enzyme action. Current research did not evaluate Lz effects on regional VMN aromatase enzyme activity or tissue estradiol concentrations as quantitative methods of requisite sensitivity for analysis of these parameters in small-volume tissue samples obtained from region-based VMN microdissection were not available. In the brain, aromatase is reportedly expressed mainly or exclusively in neurons [23]–[26]. As that published work did not include analysis of the VMN, further work is needed to characterize the VMN cell type (s) that produce neuroestradiol, and to establish how aromatase expression in that (those) cell (s) may be regulated.

Results show that during hypoglycemia, aromatase is a negative stimulus for total AMPK protein expression in the rostral, middle, and caudal VMN in female rats. As net AMPK levels in those segments did not differ after V or INS injection of V-infused females, this inhibitory tone is evidently offset by other regulatory signals at the time examined here. Since only a single time point, e.g. +1 hr post-injection was examined here, it is unclear if neuroestradiol suppression of total AMPK this sex depends upon duration of hypoglycemia. A possible physiological outcome of decreased total AMPK expression is augmentation of the cellular pAMPK/AMPK ratio, i.e. enzyme specific activity. On the other hand, diminished AMPK availability could, depending upon magnitude of decline, eventually limit enzyme mass available for activation by phosphorylation. Phosphorylation is a rapid post-translational modification that generates an appropriate acute response to hypoglycemia, whereas decrements in total AMPK protein expression may possibly serve as a more protracted adaptive response. Conversely, in the male, Lz pretreatment diminished rostral and caudal VMN AMPK levels during hypoglycemia, indicating that neuroestradiol is a positive stimulus of this protein.

Current data provide unique proof that neuroestradiol signaling is requisite for hypoglycemic activation of VMN AMPK in each sex. Interestingly, neuroanatomical patterns of VMN AMPK activation differed between sexes, as sensor activity was increased in the male rostral and middle VMN versus rostral, middle, and caudal female VMN. The neurotransmitters GABA and NO act within VMN neural circuities to correspondingly suppress or amplify counter-regulatory hormone secretion during hypoglycemia [27],[28]. Western blot analysis of pooled lysates of nitrergic and GABAergic neurons collected at regular intervals over the rostro-caudal extent of the male VMN revealed elevated pAMPK/AMPK protein ratios in each nerve cell population at a post-insulin injection time point similar to that used here [8],[9]. Present outcomes point to a need for a functional mapping approach to establish the neuroanatomical distribution of GABA and NO neurons within the VMN that exhibit neuroestradiol-dependent adjustments in neurotransmission during hypoglycemia, and to determine if hypoglycemia-sensitive cells of each transmitter type express aromatase and/or AMPK. In the hypoglycemic male, aromatase stimulation of mid-VMN pAMPK expression contrasts with negative effects of this signal on sensor activity during euglycemia. The mechanisms that cause this bi-directional metabolic state-specific neuroestradiol action remain to be clarified. It is possible that neuroestradiol may inhibit or stimulate AMPK depending upon tissue concentrations, and that local production may vary between states of glucose homeostasis versus deprivation. On the other hand, nutrient, endocrine, and neurochemical inputs to AMPK-expressing cells may modulate their receptivity to neuroestradiol. A similar premise could likely explain how hypoglycemia expands neuroestradiol regulation of AMPK activity beyond the middle VMN segment.

Electrophysiological studies have documented the presence of dedicated metabolic-sensory neurons in the VMN that supply a dynamic cellular energy readout by increasing (‘glucose-inhibited’) or decreasing (‘glucose-excited’) their synaptic firing as ambient energy substrate levels fall [29],[30]. Recent studies involving genetic manipulation of VMN AMPK α1 and −2 subunit gene expression affirm that both regulatory subunits function to promote increased electrical activity of local ‘glucose-inhibited’ metabolic-sensory neurons during hypoglycemia, but do not regulate ‘glucose-excited’ nerve cell firing [31]. Thus, current documentation of sex differences in neuroanatomical localization of hypoglycemia-associated up-regulation of pAMPK α1 and −2 subunit proteins in the VMN infer that ‘glucose-inhibited’ neurons occur throughout the rostro-caudal length of the female VMN, but are restricted to rostral and middle segments of this structure in males. Current results also provide novel evidence that ‘glucose-inhibited’ nerve cell reactivity to hypoglycemia may be neuroestradiol-dependent.

Present findings that Lz treatment prevented hypoglycemic augmentation of glucagon secretion in both sexes, and abolished hypoglycemic hypercorticosteronemia in males uniquely implicate neuroestradiol in neural mechanisms governing counter-regulation. MBH AMPK signaling is required for optimal counter-regulatory outflow [6],[7]. Evidence here for parallel Lz effects on VMN pAMPK expression and counter-regulatory hormone secretion in hypoglycemic animals suggests that these treatment outcomes may be causally associated. Nevertheless, the prospect that neuroestradiol may act outside the VMN to control counter-regulatory responses to hypoglycemia cannot be disregarded. Notably, forebrain aromatase apparently regulates hypoglycemic hypercorticosteronemia in male, but not female rats. We previously reported that *icv* ER antagonist administration alters corticosterone secretion in INS-injected rats of each sex [18],[32]. Current data suggest that the forebrain neural circuitry that controls hypoglycemic patterns of corticosterone release in females may exhibit discriminative sensitivity to systemic- versus brain-derived estradiol, by mechanisms that remain unclarified at this time.

In summary, present research establishes a role for forebrain aromatase in VMN AMPK sensor and counter-regulatory hormone responses to hypoglycemia in the rat. In each sex, neuroestradiol regulation of pAMPK expression occurs more broadly within the VMN during hypo- versus euglycemia. Outcomes show that in males, brain-derived estradiol imposes energy state-dependent control of sensor activation; further effort is required to determine how this directional, e.g. negative-to-positive shift in aromatase action between eu- and hypoglycemia occurs.
